# Resveratrol-induced brown fat-like phenotype in 3T3-L1 adipocytes partly via mTOR pathway

**DOI:** 10.29219/fnr.v64.3656

**Published:** 2020-01-14

**Authors:** Zihui Liu, Weiyao Liao, Xiaohan Yin, Xinjie Zheng, Qingrong Li, Hongmin Zhang, Lin Zheng, Xiang Feng

**Affiliations:** 1 Department of Nutrition, School of Public Health, Sun Yat-sen University, Guangzhou, China; 2 Guangdong Provincial Key Laboratory of Food, Nutrition and Health, Guangzhou, China

**Keywords:** resveratrol, 3T3-L1 adipocytes, Browning, mTOR

## Abstract

**Background:**

Browning of white adipose tissues (WAT) is recognized as a novel way to combat obesity and its related comorbidities. Thus, a lot of dietary agents contributing to browning of WAT have been identified.

**Objective:**

In this study, we try to explore the mechanism of the browning of WAT induced by resveratrol (Res) in 3T3-L1 adipocytes.

**Methods:**

The levels of cell viability and lipid accumulation were evaluated under different concentrations of Res. Cell signaling pathway analysis was performed to investigate the possible mechanisms of the WAT browning effect of Res in 3T3-L1 cells.

**Results:**

We found that Res induced the brown fat-like phenotype by activating protein expressions of brown adipocyte-specific markers, such as peroxisome proliferator-activated receptor gamma (PPAR-γ), peroxisome proliferator-activated receptor gamma coactivator-1 alpha (PGC-1α), and uncoupling protein 1 (UCP1). Besides, Res reduced lipid accumulation, as shown by Oil Red O staining. The increased small lipid droplets implied that Res-treated 3T3-L1 adipocytes had some features of brown adipocytes. The brown fat-like phenotype in 3T3-L1 adipocytes induced by Res was possibly mediated by activation of mammalian target of rapamycin (mTOR), as brown adipocyte-specific markers were decreased by rapamycin, an inhibitor of mTOR and the MHY1485 treatment, an activator of mTOR, showed the similar effect of Res on browning markers.

**Conclusions:**

Res induced brown-like adipocyte phenotype in 3T3-L1 adipocytes partly via mTOR pathway, which provided new insights into the utilization of Res to prevent obesity and related comorbidities.

## Popular scientific summary

- A new type of adipocytes, ‘beige’ or ‘brite’ adipocytes, expresses higher level of UCP1 than white adipocytes, and is morphologically like brown adipocytes.
- Resveratrol increases brown adipose tissue thermogenesis markers.
- Resveratrol influences mTORC1 or its downstream site P70S6.
- The role of mTOR in white adipose tissue browning has been paradoxical.

Obesity is a severe health problem due to its associations with various adverse health consequences, which occurs when the balance between energy intake and expenditure has been broken. In 2015, more than 100 million children and 600 million adults were diagnosed with obesity, respectively, and the prevalence of obesity is increasing year by year (1).

Two major types of adipose tissues, named white adipose tissue (WAT) and brown adipose tissue (BAT), are found in mammals, which can be discriminated by their special morphology and functions. WATs are responsible for energy storage in the form of triglycerides, whereas BATs are responsible for energy dissipation by non-shivering thermogenesis, which depends on its high content of mitochondria and expression of uncoupling protein 1 (UCP1) (2). However, the amount of BAT in adult healthy humans is quite low.

Recently, a new type of adipocytes called ‘beige’ or ‘brite’ adipocyte originated from the same progenitor as white adipocyte has been identified (3). Interestingly, this kind of adipocyte expresses higher level of UCP1 than WAT, and is morphologically like BAT, which means that beige adipocyte can increase energy expenditure as BAT.

**Fig. 1:**
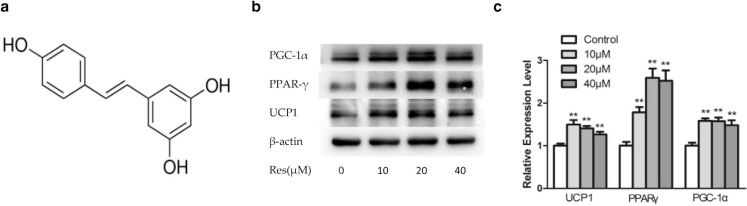
Chemical structure of resveratrol (a). Effects of Res on expression of brown adipocytes-specific markers by Western blot analysis (b) and densitometric analysis of brown adipocytes markers (c). All data are presented as the mean ± standard deviation (SD), and differences between groups were determined by one-way analysis of variance (ANOVA) using the Statistical Package of Social Science (SPSS, version 20.0; SPSS Inc.) program, followed by Tukey’s post-hoc tests. Statistical significance between control and Res-treated groups is shown as *P < 0.05 or **P < 0.01. The amount of Dimethyl sulfoxide (DMSO) was 0.08% in control.

Many natural phytochemicals such as curcumin (4), chrysin (5) and capsaicin (6) exhibit positive effects on the browning of WAT. Resveratrol (3,5,4′-trihydroxystilbene, Fig. 1a, Res) is a polyphenol existed in plants, especially in the skin of grapes, which exerts multiple physiological effects, such as antioxidant and anti-inflammatory (7), anti-carcinogenic (8), and anti-adipogenic (9) effects. Recent *in vivo* and *in vitro* studies have revealed that Res possesses the ability of increasing BAT thermogenesis markers in adipocytes (10–14). These researches suggested that Res may have potential to induce WAT browning, but the mechanism is still unclear.

Mammalian target of rapamycin (mTOR) is known as a critical regulator of cell growth and metabolism. In cell culture systems, mTOR acts as a mediator-linked nutrients and metabolic hormone signaling (15), and lately researches are revealing their roles in adipocytes *in vivo*. However, published studies on the role of mTOR in adipose tissue browning have been paradoxical. It is reported that Res influences mTOR complex 1 (mTORC1) or its downstream site P70S6 (16). In this study, we aimed to elucidate the role of Res in the formation of brown-like adipocytes in 3T3-L1 and explore mechanisms related to mTORC1 underlying this process.

## Materials and methods

### Cell culture and induction of differentiation

3T3-L1 cells were cultured in Dulbecco’s modified Eagle’s medium (DMEM) containing 10% fetal bovine serum at 37°C in a 5% CO_2_ incubator. 3T3-L1 cells were maintained in culture plates. After 100% confluence was reached, the cells were treated for 48 h with a hormone mixture containing 10 μg/mL insulin, 0.5 μM dexamethasone, and 0.5 mM IBMX (Sigma-Aldrich, MO, USA), and then exchanged with DMEM containing insulin. The cells were differentiated into adipocytes until day 8. Media was changed every 2 days. During treatments, cells were treated with different concentrations of Res for 6–8 days before further analysis. Cytotoxicity of Res was evaluated by methyl thiazolyl tetrazolium (MTT) assay as described previously and we used half-inhibitory concentration (IC50) as a measure of the potency of a substance in inhibiting a specific biological or biochemical function (17, 18).

### Immunofluorescence

Cells grown on poly-L-lysine-pretreated coverslips were fixed with 4% p-formaldehyde followed by washing with phosphate-buffered saline (PBS) and then subjected to permeabilization with 0.25% Triton X100 (Sigma-Aldrich, MO, USA). Cells were washed with PBS three times, blocked with 1% Bovine Serum Albumin (BSA) in PBS-T for 1 h, and incubated with polyclonal anti-UCP1 antibody (1:200 dilution) (Santa Cruz Biotechnology, Santa Cruz, CA, USA) overnight at 4°C, followed by three washes with PBS. Cells were then incubated with fluoresceine isothiocyanate (FITC)-conjugated antigoat secondary antibody (1:400 dilutions). 4,6-diamino-2-phenyl indole (DAPI) (Thermo Fisher Scientific, Boston, MA, USA) was used to stain nuclei of cells. Florescence images were captured using a confocal laser scanning microscope LSM700 (Carl Zeiss, Oberkochen, Germany). Analysis of images (control and curcumin-treated) was performed by software Zen 2009 (Carl Zeiss, Germany). For staining of mitochondria, Mito Tracker Red (1 mM, Cell Signaling Technology, Beverly, MA) was directly added to the growing media at a concentration of 20–25 nM, and cells were kept for 30–40 min at 37°C. After incubation, cells were fixed in 4% p-formaldehyde, followed by a single wash with PBS and immunostaining (4).

### Oil Red O staining

Control and Res-treated cells were harvested 8 days after differentiation, and then washed by PBS, fixated with 4% paraformaldehyde for 1 h at room temperature, and washed three times with double deionized water. Then cells were stained with a mixture of Oil Red O solution (0.6% Oil Red O dye in isopropanol) and water at a 6:4 ratio for 20 min and captured images under a microscope.

### Western blotting

Cells were extracted using a RIPA lysate (Beyotime, China), and then centrifuged at 12,000× *g* for 15 min. Sample were separated by 8, 10, or 12% sodium dodecyl sulfate (SDS)-polyacrylamide gel electrophoresis and transferred to a polyvinylidene difluoride membrane (Santa Cruz Biotechnology, Dallas, TX, USA), and then blocked by a solution of tris buffered saline with Tween (TBS-T) and 5% skim milk for 1 h. The membrane was rinsed by TBS-T buffer, and then incubated at 4°C overnight with 1:1,000 dilutions of primary polyclonal antibodies anti-β-actin (1:1,000; #4970; Cell Signaling Technology), anti-peroxisome proliferator-activated receptor gamma (anti-PPAR-γ) (1:1,000; #2435; Cell Signaling Technology), anti-UCP-1 (1:1,000; #14670; Cell Signaling Technology), anti-peroxisome proliferator-activated receptor gamma coactivator-1 alpha (anti-PGC-1α) (1:1,000; sc-518025; Santa Cruz Biotechnology), mTOR (1:1,000; #2972; Cell Signaling Technology), p-mTOR (1:1,000; #2971; Cell Signaling Technology), P70S6 (p70 S6 Kinase; 1:1,000; #9202; Cell Signaling Technology), and p-P70S6 (Phospho-p70 S6 Kinase; 1:1,000; #9208; Cell Signaling Technology) in TBS-T buffer containing. After washing three times, the membrane was incubated for 1–2 h with horseradish peroxidase-conjugated anti-goat immunoglobin G (IgG) or anti-rabbit IgG secondary antibody (1:1,000, Santa Cruz Biotechnology) in TBS-T buffer. Enhanced chemiluminescence (West Zol, iNtRON Biotechnology, Kyungki-Do, South Korea) was used to develop and Image J software (NIH) was used to quantify the intensities of band.

### Statistical analysis

Results were expressed as mean values ± standard deviation. The analysis of variance (two-way ANOVA) was used for statistical analysis by SPSS v.20.0 statistical analysis software (SPSS Inc., Chicago, IL, USA), followed by post-hoc tests. Values between control and Res-treated groups were considered statistically significant at either *P* < 0.05 or *P* < 0.01.

## Results

### Resveratrol increased the expression of BAT thermogenesis markers 3T3-L1 adipocytes

**Fig. 2:**
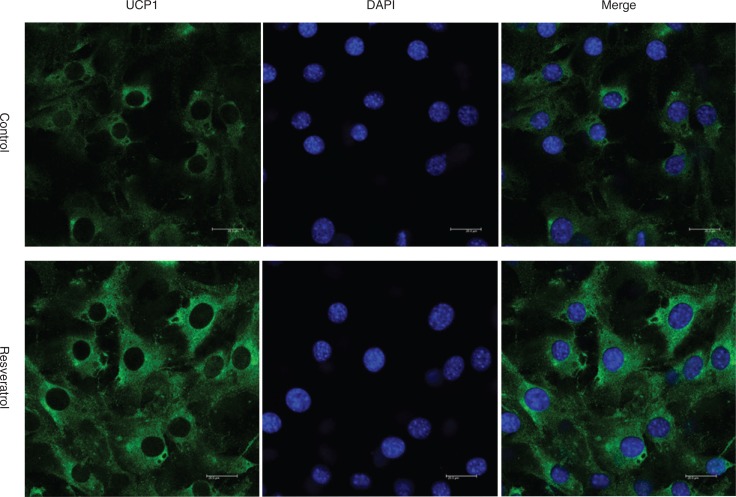
The effect of resveratrol treatment on uncoupling protein 1 (UCP1) protein expression. 3T3-L1 adipocytes with 20 μM Res treatment for 6 days were used to stain for UCP1, which were captured at 400× magnifications. The amount of DMSO was 0.08% in control.

First, cytotoxicity of Res was evaluated by MTT assay. The IC50 of Res is 48.05 μM, so we deleted the group of 80 μM. After treatment with different concentrations of Res, we found that Res synergistically increased the expression of key brown fat markers (PGC-1a, PPAR-γ, and UCP1), suggesting that white adipocytes might convert into beige adipocytes (Fig. 1b and 1c), which was confirmed by immunostaining at the cellular level (Fig. 2).

### Resveratrol-regulated lipid accumulation in 3T3-L1 adipocytes

**Fig. 3:**
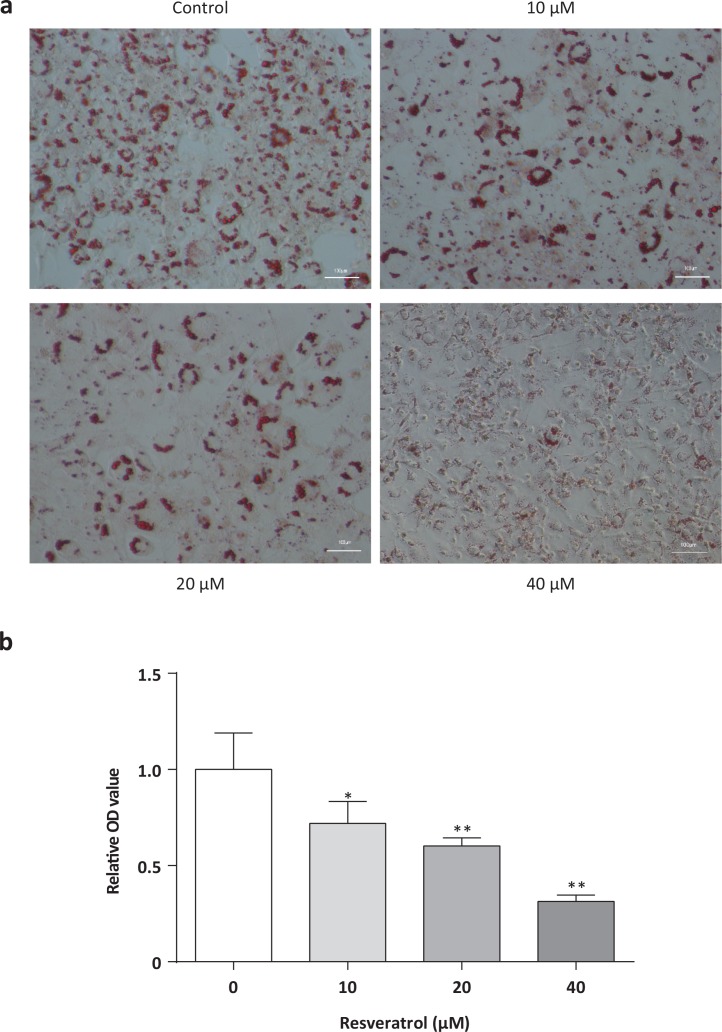
Effects of resveratrol on lipid accumulation in 3T3-L1 adipocytes. Oil Red O staining of mature 3T3-L1 adipocytes captured at 200× magnifications (a) and relative optical density (OD) value determined at OD540 (b). Oil O Red images were from 3T3-L1 adipocytes treated with Res for 8 days. All data are presented as the mean ± standard deviation (SD), and differences between groups were determined by one-way analysis of variance (ANOVA) using the Statistical Package of Social Science (SPSS, version 20.0; SPSS Inc.) program, followed by Tukey’s post hoc tests. Statistical significance between control and Res-treated groups is shown as *P < 0.05 or **P < 0.01. The amount of DMSO was 0.08% in control.

The number of small lipid droplets was increased upon Res treatment, as shown by Oil Red O staining. After removing the staining solution, the dye retained in the cells was eluted into isopropanol and OD540 was determined. The results showed that Res could reduce the lipid accumulation of adipocytes in a dose-dependent manner (Fig. 3a and 3b). These results suggested that 3T3-L1 adipocytes got some characteristic of brown adipocytes, and Res might trigger browning phenomenon in 3T3-L1 adipocytes.

### Resveratrol-induced brown adipocytes phenotype via mTOR-mediated pathway

**Fig. 4:**
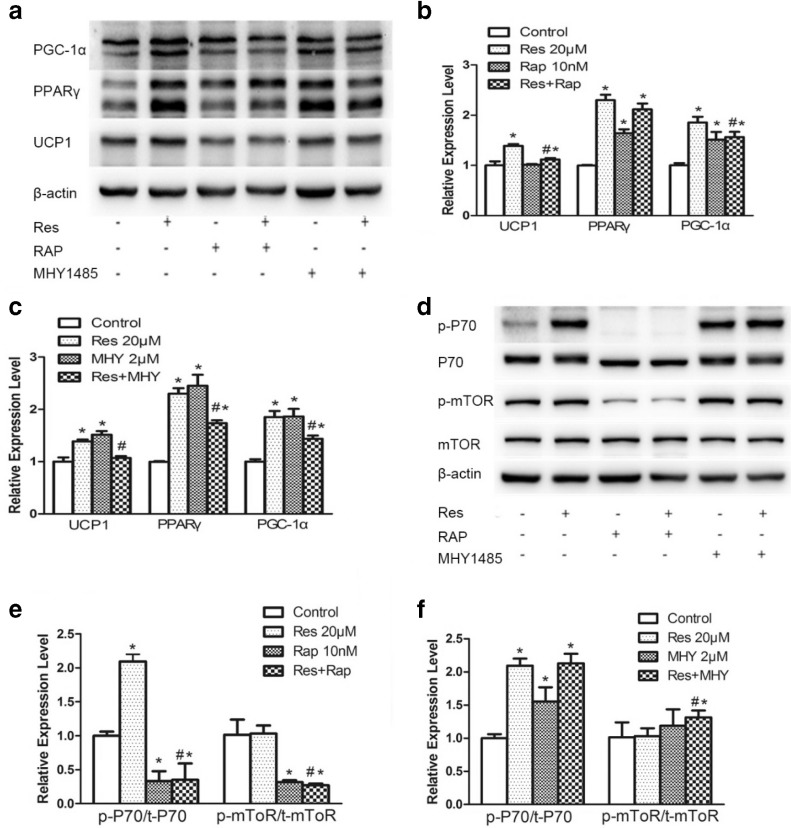
Effects of Res, rapamycin, and MHY1485 on expression of browning marker proteins via mammalian target of rapamycin (mTOR)-mediated pathway. Effects of rapamycin and MHY1485 on expression of mTOR (a) and browning marker proteins (d). The densitometric analysis of browning marker proteins (b and c) and ratio of phosphorylation to total mTOR (e and f). Rapamycin (10 nM) and MHY1485 (2 μM) were used to treat 3T3-L1 adipocytes for 6 days, and protein expression levels were determined by Western blotting. With or without treatment, each compound was indicated by ‘+’and ‘−’, respectively. All data are presented as the mean ± standard deviation (SD), and differences between groups were determined by one-way analysis of variance (ANOVA) using the Statistical Package of Social Science (SPSS, version 20.0; SPSS Inc.) program, followed by Tukey’s post-hoc tests. Statistical significance between control and Res-treated groups is shown as *P < 0.05 and between control versus other groups is shown as ^#^*P* < 0.05. The amount of DMSO was 0.08% in control.

mTOR is a key regulator of cell growth and metabolism. To explore the possible mechanism of the browning of 3T3-L1 adipocytes induced by Res, we examined expression level of mTOR. Res treatment significantly increased the expression of p-P70S6, a downstream effector of mTORC1 (Fig. 4d). Then, we treated 3T3-L1 adipocytes with a potent activator (MHY1485, 2 μM) and an inhibitor (rapamycin, 10 nM) of mTOR, respectively. Rapamycin greatly decreased the ratio of both p/t-P70S6 and p/t-mTOR (Fig. 4d and 4e). Compared to treatment with both rapamycin and Res, expression levels of UCP1, PPAR-γ, and PGC-1α treated with Res alone were also decreased (except for PPAR-γ, *P* > 0.05). The activator MHY1485 increased the expression levels of these brown marker proteins, and the tendency is similar to treatment with Res (Fig. 4a and 4c). These results suggested that Res-induced increasing expressions of brown fat markers in 3T3-L1 adipocytes might involve mTOR pathway.

## Discussion

In the present study, we found that treatment with Res enhanced the expression level of UCP1, a marker of brown adipocytes, which is consistent with the results of Wang et al. (19) in stromal vascular cells of mice. It is well established that UCP1 is located in mitochondrial inner membrane and is responsible for non-shivering thermogenesis. Shabalina et al. (20) found that UCP1 expression level in browning-isolated inguinal white adipose depots of mice almost reached that in brown adipocytes.

PPAR-γ and PGC-1α were also elevated with Res treatment. PPAR-γ is considered as a main transcriptional regulator of adipogenesis, and its role in browning has been revealed recently. Wang et al. (21) reported that PPAR-γ can induce the formation of brite adipocytes in the inguinal white adipose tissue (iWAT) depot. PGC-1α can induce uncoupling proteins and regulate nuclear respiratory factors, which, in turn, controls mitochondrial biogenesis and respiration (22). PGC-1α is identified as a transcriptional coactivator of PPAR-γ that acts at the UCP1 gene promoter (23).

There are morphological differences between white adipocytes and brown adipocytes. Brown adipocytes are polygonal cells with multilocular lipid droplets. Visibly reduction of the accumulation of lipid in adipocytes also supports this notion. So, based on the above results, we could conclude that Res can induce brown fat-like phenotype in 3T3-L1 adipocytes.

It is reported that mTOR plays a role in browning of WAT; however, it was not sure whether its function is to activate or inhibit the process of browning. Shan et al. (24) and Polak’ et al. (25) reported that adipocyte-specific mTOR-knockout or raptor-knockout decreased the weight of WATs and induced WAT-browning. However, previous studies reported that mice with mTORC1 impairment blocked the ability of β-adrenergic signaling and reduced expression of thermogenic genes in WAT (26, 27).

When it comes to the effect of Res on mTOR, the majority tend to believe that mTORC1 was inhibited by Res, especially with high concentrations (more than 50 μM) or short time treatment (<48 h) (28–31). There are also a few researchers found that Res has no effect on mTOR (32).

P70S6 is one of the best characterized downstream effector of mTORC1, which can phosphorylate and activate the 40S ribosomal S6 protein. Armour et al. identified S6K1 as the direct target of Res (33). Demidenko et al. found it is possible that low concentrations (<12.5 μM) of Res increase S6 phosphorylation in HT-p21 cells (30). Besides, Huang et al. reported that 0.2–2 μM treatment in 2BS and WI38 cells can activate phosphorylation of S6K1 (34). In our study, after 8 days of 20 μM Res treatment, the phosphorylation of S6K1 was enhanced. Rapamycin successfully blocked mTOR and eliminated the stimulating effects of Res on the browning markers, and MHY1485 treatment mimicked the effects of Res on browning markers, reinforcing our assumption that Res induced browning via the mTOR-mediated pathway.

## Conclusion

In conclusion, our findings suggest that Res plays a dual modulatory role in the form of inducing the brown-like phenotype, and mTOR pathway was involved in this process. So, Res may be explored as a potentially promising food additive for prevention of obesity. However, the detailed mechanism that underlies these effects remains unclear and studies *in vivo* are required to ascertain its role.

## Data Availability

The data sets used and/or analyzed during this study are available from the author (Weiyao Liao) on reasonable request. Please contact author (Weiyao Liao) for data or material requests.
